# Plantar pressure changes in hindfoot relief devices of different designs

**DOI:** 10.1186/s40634-019-0173-9

**Published:** 2019-02-07

**Authors:** F. Mazur, B. Swoboda, H. D. Carl, C. Lutter, M. Engelhardt, M. W. Hoppe, T. Hotfiel, C. Grim

**Affiliations:** 1 Division of Orthopaedic Rheumatology, Department of Orthopaedic Surgery, Friedrich-Alexander-University Erlangen-Nuremberg, Rathsberger Str. 57, D-91054 Erlangen, Germany; 2 Department of Orthopaedic and Trauma Surgery, Martha-Maria Hospital, Nuremberg, Germany; 3 Department of Orthopaedic and Trauma Surgery, Sports Orthopaedics and Sports Medicine, Klinikum Bamberg, Bamberg, Germany; 4 Department of Orthopaedics, Trauma and Hand Surgery, Klinikum Osnabrück, Osnabrück, Germany; 5 Department of Movement and Training Science, University of Wuppertal, Wuppertal, Germany

**Keywords:** Plantar pressure, Hindfoot relief shoes, Plantar ulcers, Kinetics, Biomechanics, Pedobarography, Foot, Stress fractures

## Abstract

**Background:**

It is frequently observed that overloading the foot can impair bone and soft tissue healing and can lead to harmful sequelae (i.e. ulcers, stress reactions) in context of pre-existing tissue disabilities. In terms of offloading, hindfoot relief devices are commonly applied as a non-operative treatment as well as after various surgical procedures for hindfoot disorders. Despite their common use, there is a paucity of data comparing different orthotic devices with respect to changes in plantar pressure distributions. The aim of this study was to investigate plantar loadings in hindfoot relief devices of different designs.

**Methods:**

Twenty-five healthy participants (13 women, 12 men; (mean ± SD) age 37 ± 14 years; BMI 23 ± 4 kg/m^2^) were recruited. Plantar pressure distributions were collected using i.) a neutral shoe, ii.) a hindfoot relief shoe (HRS) and iii.) a hindfoot relief orthosis (HRO). Peak pressure values were measured via dynamic pedobarography during walking and were analysed from four different plantar regions: the hindfoot, midfoot, metatarsal I-V and forefoot. As a reference standard, the normal walk using neutral shoes served as the condition for full weight-bearing.

**Results:**

Concerning the hindfoot, using the HRS as well as the HRO resulted in significant decreases in plantar pressures compared to baseline values that were obtained with the neutral shoe (− 52% for the HRS and − 52% for the HRO, *p* < 0.001). Significant increases in peak pressures were found in the midfoot region for both devices (HRS: 32%, *p* = 0.002; HRO: 47%, *p* < 0.001). For the metatarsal region, peak pressures were found to decrease significantly (HRS: − 52%, *p* < 0.001; HRO: -17%, *p* = 0.034). With respect to the forefoot, a significant reduction in peak pressures using the HRS (− 41%, *p* < 0.001) was detected, whereas the HRO did not lead to significant changes (− 4%, *p* = 0.691).

**Conclusions:**

Both the HRO and HRS significantly reduced plantar hindfoot pressure, corresponding to a relative decrease of nearly 50% of the baseline. Nevertheless, the adjacent midfoot zone displayed a significant increase in plantar pressure values for both devices. Supported by these findings, physicians should cautiously consider a substantial increase in midfoot loading, especially in patients affected by additional midfoot injuries or accompanying impairments of tissue healing.

**Level of evidence:**

IV, Case series.

## Background

The concept of offloading the foot has been established as a treatment strategy after various surgical and non-operative procedures in the context of trauma, illnesses and disabilities of the foot and ankle (Bus et al., 2016; Bus & Valk, 2008; Baur et al., 2018). It is frequently observed that overloading the foot accompanied by elevated pressure pattern are thought to have important roles for the development of impairments of wound and softtissue healing or can cause delays in fracture healing (Claes & Heigele, 1999; Reike et al., 1997; Genc et al., 2016). Hindfoot relief devices have commonly been used in the post-surgical rehabilitation process, following various procedures such as the repair of calcaneal fractures, ligament reconstructions, corrective osteotomies, and trauma surgery of the hindfoot (Carl et al., 2006; Hodge et al., 1999; Schepers et al., 2008; Bohl et al., 2017; Groot et al., 2013; Cavanagh & Bus, 2011; Kraus et al., 2014; Bus et al., 2009). In cases of tarsal bone marrow oedema, stress reactions or stress fractures, hindfoot relief devices allow a mobilization under limited weight-bearing conditions that are encouraged to promote healing without overloading the tissue (Pauser et al., 2011). Additionally, hindfoot relief devices are used to improve the healing process for plantar ulcers and wound healing disorders due to trauma, peripheral arterial disease, neuropathic disabilities and rheumatoid arthritis (Pauser et al., 2011; Götz et al., 2016; da Conceição et al., 2015). Offloading the hindfoot is mostly carried out by hindfoot relief shoes (HRSs) and hindfoot relief orthoses (HROs) (Hunt et al., 1987; Hahn et al., 2014). Nevertheless, commonly available devices display fundamentally different designs and concepts. Despite the common use of pressure relief devices, there is a paucity of data comparing their offloading effects related to biomechanical aspects.

Dynamic pedobarography is a modality that has been widely validated as a method to evaluate plantar pressure under dynamic conditions (Skopljak et al., 2014). Owing the ability to record consecutive steps in one measurement, insole-based pedobarography has become an important tool for the evaluation of foot loads during the application of insoles, orthoses or other types of footwear (Skopljak et al., 2014; Westphal et al., 2016; Kluger et al., 2014; Lorkowski et al., 2015). By this approach, the offloading effects of forefoot relief shoes in surgical or non-surgical terms have been extensively investigated (Carl et al., 2006; Kraus et al., 2014; Bus et al., 2009). In contrastthere has been a paucity of data comparing plantar pressure patterns in HRSs of various designs (Hahn et al., 2014). To our knowledge, there has been no study assessing foot load pattern in HRSs in comparison to HROs. Knowledge regarding the resulting loads during the rehabilitation and healing processes are nevertheless of high clinical interest. We focused on mean peak pressure pattern via dynamic pedobarography in an HRO and an HRS. As a reference standard the normal walk using neutral shoes served as the condition for full weightbearing.

## Materials and methods

### Study population

Twenty-five healthy volunteers were enrolled (13 women, 12 men; mean age 47 ± 14 years; mean BMI 23 ± 4 kg/m^2^) with no signs of foot or lower limb complaints. Exclusion criteria were any history of lower limb surgery, significant leg length discrepancy, lower limb malalignment or history of acute or overuse injuries of the lower limb.

Every participant was examined according to full range of ankle motion and ankle stability. Two volunteers were excluded from the analysis, as they did not fulfil the inclusion criteria (one participant had a lateral ankle instability; one presented midfoot pain).

### Data acquisition

**Fig. 1:**
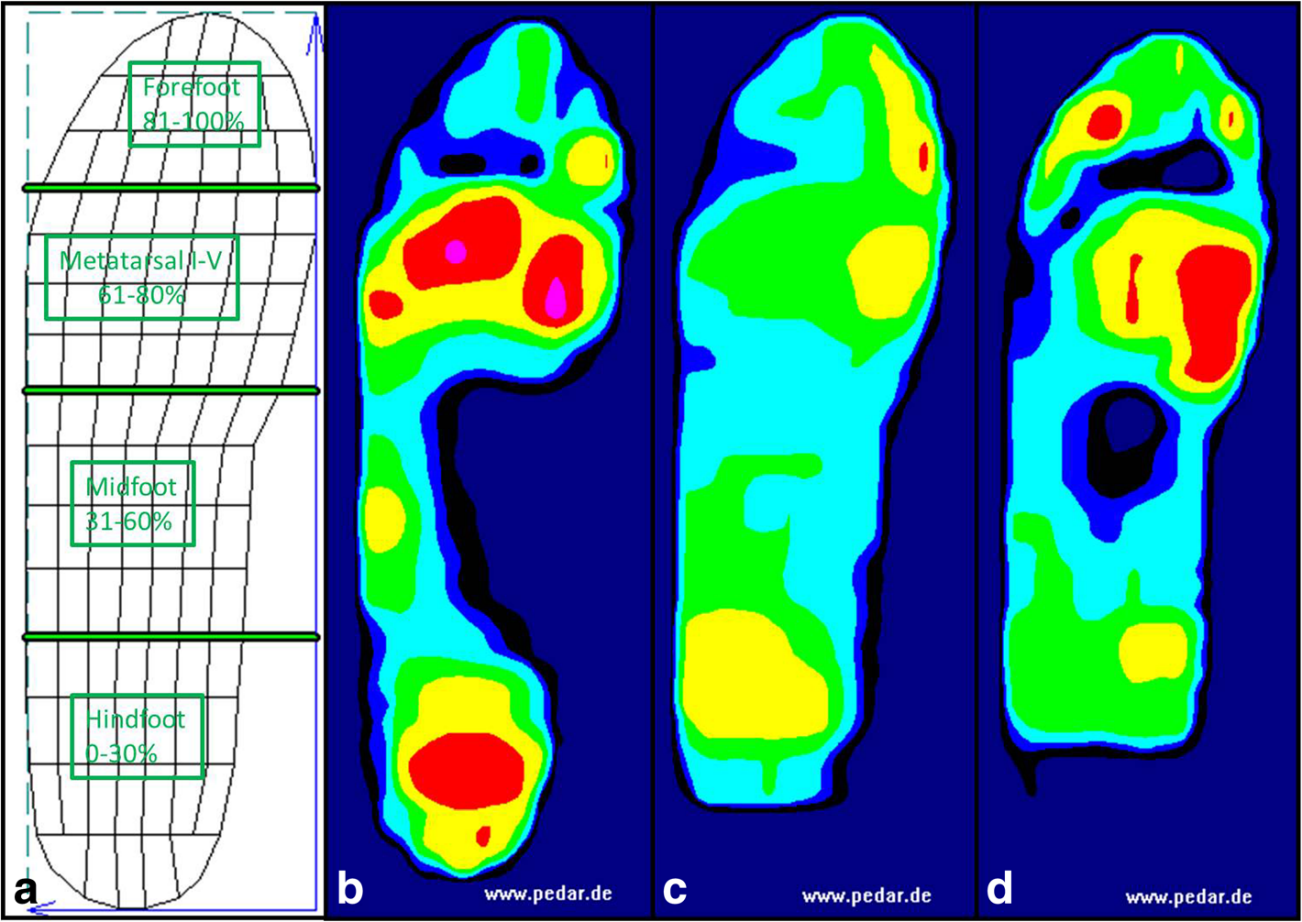
Demonstrating the subdivision of the plantar surface into four anatomical regions (**a**). Exemplary graphical illustration of mean peak pressure values assessed on the different devices (**b-d**); **b**: neutral shoe; **c**: hindfoot relief shoe; **d**: hindfoot relief orthosis

Pedobarographic data were obtained using the pedar-X system (novel GmbH, Munich, Germany), consisting of insoles holding 99 separate pressure sensors that operate at a frequency of 50 Hz. Peak pressure values (kPa, highest values during each step and region) were obtained from 12 steps per foot during walking, following previously published protocols (Arts & Bus, 2011). The sizes of the measurement insoles were adjusted individually based on each participant’s foot size. The plantar foot was subdivided into four anatomical regions (Westphal et al., 2016), representing the hindfoot (0–30% length, 0–100% width), midfoot (31–60% length, 0–100% width), metatarsal I-V (61–80% length, 0–100% width) and forefoot (81–100% length, 0–100% width) (Fig. 1). A total of three trials, each with different devices, were performed. Measurements were taken indoors on a level surface, while walking speeds were kept constant at 3.5 km/h using a photo-barrier (Baur et al., 2018; Burnfield et al., 2004).

**Fig. 2:**
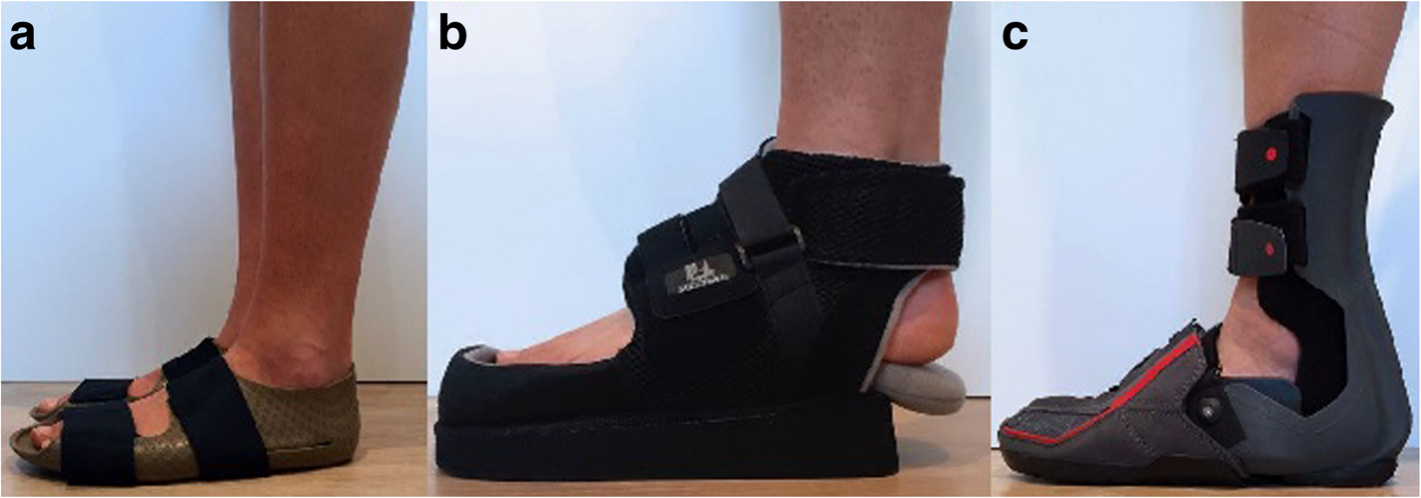
**a**: Illustration of the neutral shoes that were used to assess the reference values for dynamic pedobarography (Fuss und Schuh Breidbach® Inc., Fulda, Germany). **b**: Illustration of Hindfoot relief shoe (HRS) (München, Fior and Gentz Inc., Lueneburg, Germany®) and **c**: Illustration of Hindfoot relief orthosis (HRO) (“Dr. Settner/Münch”, Otto Bock Health Care Germany GmbH (OBHCD)®)

In advance of every trial, the volunteers performed a 10–15 min walk to become accustomed to each device. During the first trial the participants were asked to walk with a neutral shoe (Fuss und Schuh Breidbach® Inc., Fulda, Germany) (Fig. 2a) to define baseline values and equalize conditions of full weight-bearing. The shoe was established as a reference shoe for dynamic pedobarography (Kluger et al., 2014). It is composed of 4 mm polyethylene-vinyl acetate and has a heel pitch of 0 mm; elastic velcro buckles allow adjustment and fixation around the foot.

The second trial was performed wearing an HRS (München®, Fior and Gentz Inc., Lueneburg, Germany) (Fig. 2b). The plantar hindfoot relief zone of this device is approximately 20% of the whole sole length. This shoe consists of a wedge-designed sole with a 5° slope and measures approximately 5 cm at the highest point. The sole is made of polyethylene-vinyl acetate. The symmetrical shoe can be used for both the left and right sides.

The third trial utilized an HRO (“Dr. Settner/Münch”®, Otto Bock Health Care Germany GmbH (OBHCD)) (Fig. 2c). This orthosis is based on a modular system that allows a customized individual adaption. The relief zone is approximately 25–30% of the entire foot length. The HRO incorporates an outsole thickness of 1 cm height. It is based on a modular system and includes a further inner insert of approximately 4 cm peak height (peak heights are exemplary given for size “L”). For both devices, the size was adjusted individually based on the manufacturers’ instructions.

During the second and the third trial a conventional available running shoe (The Faas 500, Puma Inc., Herzogenaurach, Germany), categorised as a “neutral running shoe”, was applied at the contralateral side (Kluger et al., 2014). According to the manufacturer this shoe has no pronation or supination support. For each participant only one foot was determined for data analyses (Vette et al., 2019; Gray et al., 2014).

### Statistical analysis

For each participant kinetic data were computed as the mean peak pressure value for each specific region (mean value of each trial) using the novel multiprojects-ip software package (Novel GmbH, Munich, Germany). Within the defined specific region, the sensor with the highest value was representative for each stance phase and averaged for the series of 12 steps.

Data were then transposed to Prism 7 software (GraphPad Software Inc., San Diego, California®). Data were verified for normality with the D’Agostino Pearson test. In case of normality, the paired t-test was used. Otherwise the Wilcoxon matched-pairs signed rank test was applied to compare the HRS and HRO to control conditions as well as to each other. *P*-values < 0.05 were regarded as statistically significant.

### Ethics approval and consent to participate

The local Ethics Committee approved the study with no requirements (Ref. No. 57_17 B; University of Erlangen-Nuremberg). All patients were informed regarding the purpose, benefits and risks of the investigation prior to signing an institutionally approved informed consent form to participate in the study.

## Results

**Fig. 3:**
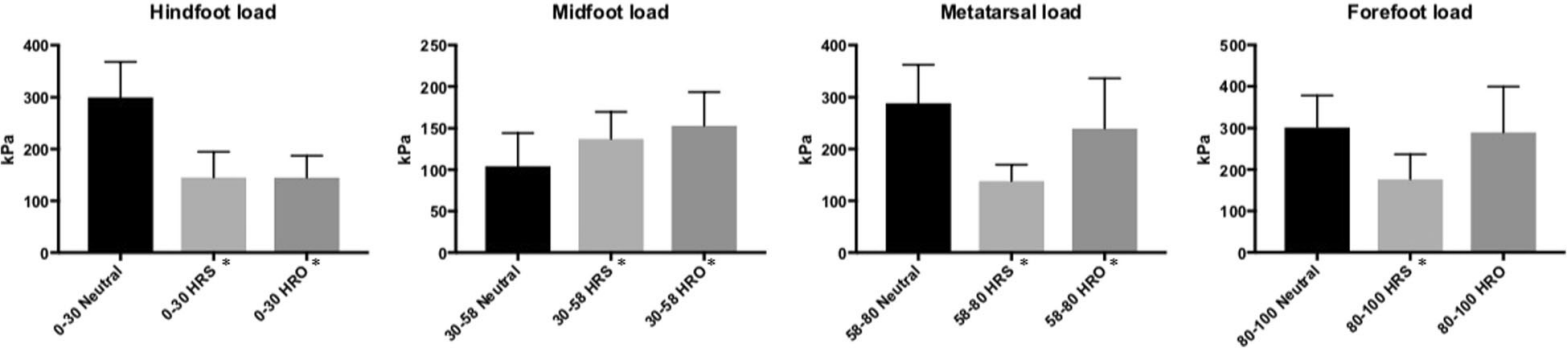
Bars illustrate peak pressure values (kPa) under the hindfoot, midfoot, metatarsal zone and forefoot for the different devices (neutral shoe, HRS and HRO). *: Statistically significant difference to neutral conditions

Descriptive results are listed in Table 1 and graphically illustrated in Figs. 1 and 3.

**Table 1:** Absolute peak pressure values in kPa (mean ± SD) for the described anatomical regions and percentage changes compared to baseline

kPa	Neutral Shoe	Hindfoot relief shoe - HRS	Hindfoot relief orthosis – HRO
Hindfoot	300 ± 68	145 ± 50 (-52%; *p* < 0.001)	145 ± 43 (-52%; *p* < 0.001)
Midfoot	104 ± 40	137 ± 33 (+32%; *p* = 0.02)	153 ± 41 (+47%; *p* < 0.001)
Metatarsal Zone	288 ± 74	138 ± 32 (-52%; *p* < 0.001)	240 ± 96 (-17%; *p* = 0.034)*
Forefoot	302 ± 77	177 ± 60 (-41%; *p* < 0.001)	290 ± 110 (-4%; *p* = 0.692)*

Peak pressure values (kPa) mean ± SD for all foot regions and percentage change compared to the Neutral shoe

*Statistically significantly difference comparing the HRO and the HRS

### Neutral shoe

Peak pressure values (mean ± SD) obtained in the neutral shoe were 300 ± 68 kPa under the hindfoot, 104 ± 40 kPa under the midfoot, 288 ± 74 kPa under the metatarsal zone and 302 ± 77 kPa under the forefoot. Concerning the entire foot, a pressure value of 346 ± 66 kPa was measured.

### HRS and HRO

#### Hindfoot

The HRS revealed 145 ± 50 kPa, indicating a statistically significant reduction in hindfoot peak pressure of 52% in comparison to the baseline value obtained in the neutral shoe (*p* < 0.001). The HRO showed a reduction in hindfoot peak pressure of 52% (145 ± 43 kPa) which was also significantly different from the baseline (*p* < 0.001). The HRO and HRS peak pressures were not significantly different (*p* = 0.960).

#### Midfoot

Concerning the midfoot, peak pressure values of 137 ± 33 kPa for the HRS, indicate a significant increase in comparison to the baseline (132% baseline value; *p* = 0.002). HRO values of 153 ± 41 kPa were obtained, indicating a 147% increase (*p* < 0.001). Values were not significantly different between each device (*p* = 0.120).

#### Metatarsal

Metatarsal zone peak pressures were significantly lower with the HRS as well with the HRO (138 ± 32 kPa; *p* < 0.001 and 240 ± 96 kPa; *p* = 0.034, respectively). The comparison between the HRO and the HRS revealed a significant reduction for the HRS compared with the HRO (*p* < 0.001).

#### Forefoot

Regarding the forefoot, the HRS had a significant reduction in peak pressure to 59% of the baseline value (177 ± 60 kPa; *p* < 0.001). The HRO showed 96% of the baseline value (290 ± 110 kPa), which was not significantly different from the baseline; *p* = 0.692); HRO values were significantly different from those of HRS (*p* < 0.001).

## Discussion

Despite the wide use of HRS and HRO in clinical practice, there is a paucity of data representing biomechanical changes of plantar pressure distribution using commonly applied offloading devices, and outcomes are even less often investigated. It is hypothesized that the clinical effects of hindfoot relief orthoses are based on offloading effects to the plantar tissue (Hahn et al., 2014). Nevertheless, to date, no study has compared such biomechanical tissue responses between HRO and HRS. Offloading effects of forefoot relief devices are already benchmarked and well-studied and have helped to transfer biomechanical principles to clinical implications (Bus et al., 2016; Cavanagh & Bus, 2011; Bus et al., 2009). To our knowledge, the present study is the first to assess plantar pressure distributions via dynamic pedobarography in hindfoot relief devices of various designs comparing data to conditions of full weight-bearing. Moreover, for the first time we demonstrated a hindfoot peak pressure reduction with an HRO.

Our study revealed several main findings. First, we demonstrated significant offloading effects for the hindfoot area, and second, we observed significantly elevated peak pressures for the adjacent midfoot region both for the HRO and HRS.

Surprisingly, our study demonstrated that there were no significant differences among hindfoot relief devices of different designs. The decrease in the plantar pressure to the hindfoot that was observed for the HRS may be explained by the midsole concept that represents a 5° sloped, wedge-designed hindfoot relief zone. Offloading effects of the HRO may be achieved by the lever-type shaft construction. Thus, both devices display fundamentally different offloading concepts. Nevertheless, their offloading effects were nearly similar (*p* > 0.05), and we cannot recommend one or the other of these devices based on the offloading effects. However, there were significant differences corresponding to the metatarsal and forefoot region. Our results showed significantly higher peak pressure reductions in the metatarsal and forefoot region for the HRS (− 52% and − 41%) compared to the HRO (− 17% and − 4%). Nevertheless, no device displayed elevated values in comparison to baseline. Hindfoot peak pressure reductions of nearly 50% of the baseline obtained in this study were comparable to those of a previous investigation by Hahn et al., who evaluated different types of HRSs (Hahn et al., 2014). The authors reported a decrease of hindfoot load of 90% (0–15% of sole) and 18% (15–30% of sole) for the devices used in the study (Hahn et al., 2014). A weakness of that study was that HROs were not included. With respect to the offloading effects of forefoot relief shoes (FRS), peak pressure reductions of 38 to 58% have been reported (Bus et al., 2009). Previous studies and reviews by Bus et al. have already provided evidence of forefoot offloading concepts concerning ulcer prevention (Bus & Valk, 2008; Bus, 2016). Based on our results we confirmed the significant peak pressure reduction using an HRS observed by Hahn et al. with comparable values (Hahn et al., 2014).

### Clinical implications

Although limited weight-bearing is often required by surgeons’ specifications, there have been no evidence-based rehabilitation guidelines that determine exact values of weight-bearing graduations in accordance with operative or non-operative interventions (Wild et al., 2016). In rehabilitation after lower limb surgery there is a lack of unified, evidence-based rehabilitation concepts (Pfeifer et al., 2015). However, if total offloading to the hindfoot is required, our results indicate that neither HROs nor HRSs are able to alleviate plantar pressure at all, as 50% of the baseline must be considered. Furthermore, increased midfoot load must be cautiously considered. Our data indicated significant peak pressure increases at the midfoot region while using hindfoot relief devices. Similar pressure shifts have been described for forefoot relief devices (Mueller et al., 2016; Cousins et al., 2013; Birtane & Tuna, 2004). Regarding the HRS (München shoe), Hahn et al. reported only a 5% increase, whereas our study demonstrated a significant 32% increase for the midfoot region. An increased midfoot vulnerability using orthotic devices was already reported for an ankle-foot orthosis (Vacoped®) by Pauser et al. (Pauser et al., 2012). Based on the existing investigations identifying increasing plantar pressure in the midfoot, as in our findings, the midfoot area appears to be a sensitive area for adapting increasing foot loads (Hotfiel et al., 2017). Regarding the localization of stress fractures, to which peak pressure is a commonly accepted risk factor, the midfoot area displayed the highest incidence in contrast to the tarsal bone, sesamoid or toe phalanx (Hotfiel et al., 2017). In particular, patients with accompanying midfoot injuries or neuropathic or diabetic diseases should undergo a regular clinical examination to avoid further damage.

Interestingly our results offered differences in peak pressure patterns between the HRS and HRO in regard to the metatarsal and forefoot region. Hence clinical implications should be considered here as well. The significant lower peak pressure using the HRS could be relevant for patients with simultaneous complaints to the metatarsal or forefoot regions.

Considering the clinical relevance in view of plantar ulcers or stress fractures, loaded body weight must be seen as risk factor for these groups. Our data highlighted that dynamic pedobarography may routinely be applied to assess elevated plantar pressure pattern if hindfoot relief shoes are to be prescribed. Studies have demonstrated peak pressure reduction in the forefoot using cushioning pads (Baur et al., 2018). Further studies are required to evaluate whether cushioning or individual modifications are useful to compensate for elevated midfoot loads particularly in hindfoot relief devices.

### Pedobarography

Dynamic pedobarography was chosen for the assessment of foot loading because it has been established as a useful adjunct to clinical research for the recognition of plantar pressure conditions (Baur et al., 2018; Westphal et al., 2016; Hahni et al., 2016; Mehlhorn et al., 2017). Hindfoot weight-bearing was defined as a limitation of loads on the plantar surface, assessed by dynamic pedobarography. Although this definition is widely accepted, there is no clear-cut evidence that foot load actually is a sufficient surrogate parameter for weight-bearing conditions in regard to intraosseous or intraarticular loading (Wild et al., 2016; Schaefer et al., 2015). We decided to assess peak pressure in accordance with the vast majority of previous investigations evaluating plantar loading under various conditions (Hotfiel et al., 2017).

## Study limitations

This study has few limitations. First our results do not allow statements regarding estimation of gait stability or comfort while using the orthotic devices. Pain or discomfort could be a trigger for unintentional overload of the contralateral foot. In this context, we did not assess kinematic data of the hip, knee and ankle to observe in which position of the gait cycle peak pressures develop. Altered biomechanics of the limb may play a role in the change of foot loading. Second, our study was comprised of healthy participants and not patients. When designing the study, we could not rule out the possibility that some settings exceeded a certain limitation of weight bearing, and patients might have been jeopardized. However, future investigations including selected patients (injuries as well as pre-existing disabilities), are needed to confirm findings which were obtained in this study. In these studies further functional kinetic parameters (i.e. normal impulse-based measures (Vette et al., 2019)), that may provide differential information on loading should be implemented too.

## Conclusions

Taken together, our results suggest that hindfoot relief shoes and orthoses significantly decrease plantar peak pressure to the hindfoot. There was no significant difference between the HRO and HRS. In terms of offloading, we cannot recommend for or against using an HRO or an HRS. Nevertheless, the reduction of hindfoot pressure was accompanied by a significant increase of midfoot load. This finding could be of high clinical relevance in the context of underlying midfoot injuries or impaired conditions of tissue healing.
